# Cardiopulmonary Resuscitation Pattern Evaluation Based on Ensemble Empirical Mode Decomposition Filter via Nonlinear Approaches

**DOI:** 10.1155/2016/4750643

**Published:** 2016-07-26

**Authors:** Muammar Sadrawi, Wei-Zen Sun, Matthew Huei-Ming Ma, Chun-Yi Dai, Maysam F. Abbod, Jiann-Shing Shieh

**Affiliations:** ^1^Department of Mechanical Engineering and Innovation Center for Big Data and Digital Convergence, Yuan Ze University, Taoyuan, Chung-Li 32003, Taiwan; ^2^Department of Anesthesiology, College of Medicine, National Taiwan University, Taipei 100, Taiwan; ^3^Department of Emergency Medicine, College of Medicine, National Taiwan University, Taipei 100, Taiwan; ^4^Graduate Institute of Networking and Multimedia, National Taiwan University, Taipei 100, Taiwan; ^5^Department of Electronic and Computer Engineering, Brunel University London, Uxbridge UB8 3PH, UK

## Abstract

Good quality cardiopulmonary resuscitation (CPR) is the mainstay of treatment for managing patients with out-of-hospital cardiac arrest (OHCA). Assessment of the quality of the CPR delivered is now possible through the electrocardiography (ECG) signal that can be collected by an automated external defibrillator (AED). This study evaluates a nonlinear approximation of the CPR given to the asystole patients. The raw ECG signal is filtered using ensemble empirical mode decomposition (EEMD), and the CPR-related intrinsic mode functions (IMF) are chosen to be evaluated. In addition, sample entropy (SE), complexity index (CI), and detrended fluctuation algorithm (DFA) are collated and statistical analysis is performed using ANOVA. The primary outcome measure assessed is the patient survival rate after two hours. CPR pattern of 951 asystole patients was analyzed for quality of CPR delivered. There was no significant difference observed in the CPR-related IMFs peak-to-peak interval analysis for patients who are younger or older than 60 years of age, similarly to the amplitude difference evaluation for SE and DFA. However, there is a difference noted for the CI (*p* < 0.05). The results show that patients group younger than 60 years have higher survival rate with high complexity of the CPR-IMFs amplitude differences.

## 1. Introduction

Cardiac disease and out-of-hospital cardiac arrest (OHCA) are the major healthcare problem internationally [1, 2]. Despite advances in medicine and cardiology, OHCA is still associated with a high mortality rate [3, 4]. One of the main causes of OHCA is severe ischemic heart disease, including the acute coronary artery occlusion [5–7]. According to Eisenberg et al., successful return of spontaneous circulation (ROSC) from OHCA is based on certain factors, such as the general condition of the patients, the type and vitality of the events, and the duration to bystander cardiopulmonary resuscitation (CPR) being delivered [8].

CPR is one of the fundamental links in the chain of survival in the management of the OHCA patients. When the connections between each other are well performed, the survival rate will increase significantly [9]. On the other hand, the unexpected cardiac rhythm can be escalated when one of these connections is postponed [10, 11]. An effective chest compression itself involves the application of the pressure to the sternum maintaining the flow of blood and oxygen to myocardium and brain [12]. The chest compression condition is a dominant index of the CPR accomplishment [13–15]. In order to evaluate the CPR data, the noise is an essential concern. A filtering method can be performed in order to extract the correct information from the continuous signal. The use of empirical mode decomposition (EMD) filtering algorithm, proposed by Huang et al. [16, 17], has been used to filter signal problems, such as EMD-based filters which have also been used for narrow-band signals such as electrocardiography (ECG) [18] and blood pressure [19].

In advance, the filtered signal is extracted to achieve the information containing its characteristics. One of these methods, the entropy algorithm, was used in information theory [20] to address the nonlinearity problems. An entropy algorithm was also applied to the ECG signal studies [21, 22]. In a study by Costa et al., extended sample entropy was applied to evaluate the feature extraction of the ECG using multiscale entropy [23]. Another nonlinear method, detrended fluctuation analysis (DFA), was originally utilized for the DNA sequence [24].

Studies related to purifying the signal and extracting information for the cardiac arrest cases have been done for several years. A study utilizing a multichannel Wiener filter and a matching pursuit-like method is conducted to remove CPR artifact from the ECG trace [25]. Least mean-square (LSM) filtering has also been utilized to remove the CPR problem [26]. A new method combining the noise-assisted multivariable EMD (N-A MEMD) and LSM filtering was implemented by Lo et al. [27]. Furthermore, the application of the sample entropy has been utilized for shock outcome prediction [28] as well as multiscale entropy [29]. Detrended fluctuation analysis was utilized by Lin et al. for the study of ventricular fibrillation in OHCA cases [30]. The purpose of this study is to evaluate the CPR pattern by utilizing the EEMD to purify the CPR signal and the ECG data by applying the nonlinear algorithms to see the survival rate.

## 2. Data Acquisition and Algorithm

### 2.1. Data Acquisition

The dataset is retrospectively collected from the New Taipei City fire-based of emergency medical service (EMS). All the staff have been trained for the basic life support, early defibrillation, and advanced life support. All the ambulance units are equipped with a ForeRunner AED (Philips, Seattle, WA, USA). The ECG signal is logged into the AED card data, sampled for 200 Hz. The logging lead was placed on the patient chest [27].

**Figure 1:**
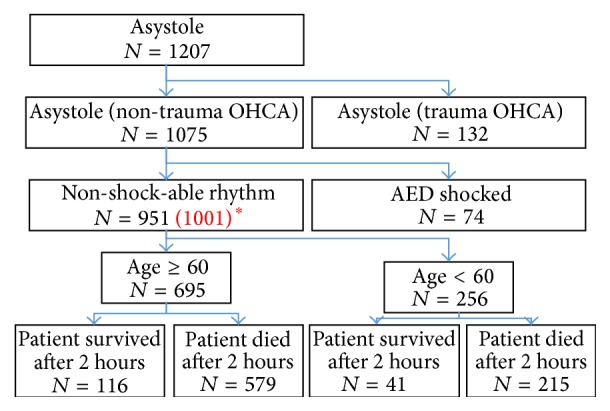
The flowchart of the CPR evaluation. ^**∗**^ *Note*. The original 1001 ECG signals have to be reduced due to the quality of the data.

This study has utilized data from the whole year of 2010. A total of 1207 patient ECGs are divided into two groups: trauma and non-trauma cardiac arrest. Focusing on the non-trauma patients only, the data is divided into another two groups: patients who had an AED shock and non-shock-able signal patients. In order to evaluate the pure CPR without any help of the AED, all the 1001 non-shock-able patients, which eventually becomes 951 sets after filtering for the quality of the data, are divided according to their age with the threshold of 60 years, as shown in Figure 1. The outcome of the patient is evaluated after 2 hours based on their conditions. The results are analyzed in MATLAB language (Mathwork Inc.).

### 2.2. Empirical Mode Decomposition-Based Filter

#### 2.2.1. Empirical Mode Decomposition

EMD is an algorithm to decompose the specific frequency range of the data into a finite number of intrinsic mode functions (IMFs). These decomposed IMFs illustrate certain characteristics. However, for the real-world signals, the mode-mixing disturbs the regularity of the IMFs. For this reason, the ensemble empirical mode decomposition (EEMD) was proposed.

#### 2.2.2. Ensemble Empirical Mode Decomposition

The intermittence corrupts the consistence of the IMFs. The subsequent mode function will be affected, hence the physical meaning of those IMFs that cannot be parted based on their characteristics. Wu and Huang [31] proposed EEMD using noise-assisted method to overcome this phenomenon. In EEMD, the white noise is added to the original signal to form a mixed combination of noise and signal in order to remove the intermittence and generate consistent IMFs. EEMD study was also conducted to an ECG noise filtering problem [32].

### 2.3. Feature Extraction Algorithms

#### 2.3.1. Sample Entropy and Complexity Index

Entropy is known as a thermodynamics property in the evaluation of regularity. The higher the entropy means the less regular the pattern or the sequence to be recognized. For the multiscale entropy, the coarse grained time series is based on the scale factor [23]. The coarse grained time series will be evaluated by entropy algorithm. The result of the entropy corresponds to each scale which is called multiscale entropy. The complexity index (CI) is defined as measurement of the signal complexity. It is calculated by the evaluation of the area under curve of the multiscale entropy. The calculation from the recreated time series based on the coarse grained information will affect the area under the area of the curve.

#### 2.3.2. Detrended Fluctuation Analysis

Fractal analysis is one of the most prosperous methods to get the signal features. Detrended fluctuation analysis (DFA) is a nonstationary algorithm for statistical analysis. A considerably physiology-related problem is a nonstationary time series one. This method is originally proposed by Peng et al. [24].

## 3. Results and Discussion

**Figure 2:**
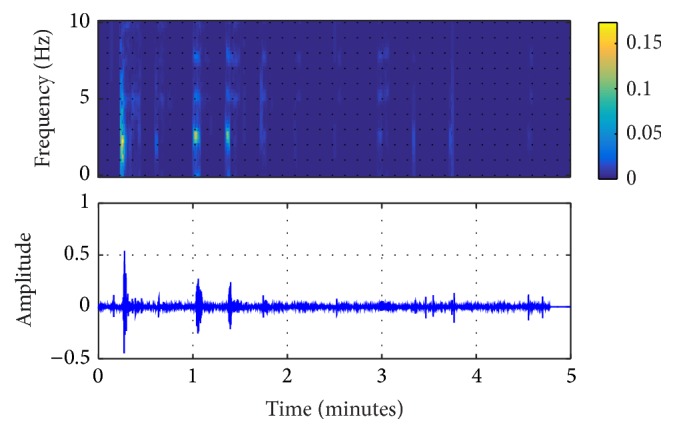
EEMD-extracted CPR and the time-frequency information of IMF 2.

**Figure 3 fig3:**
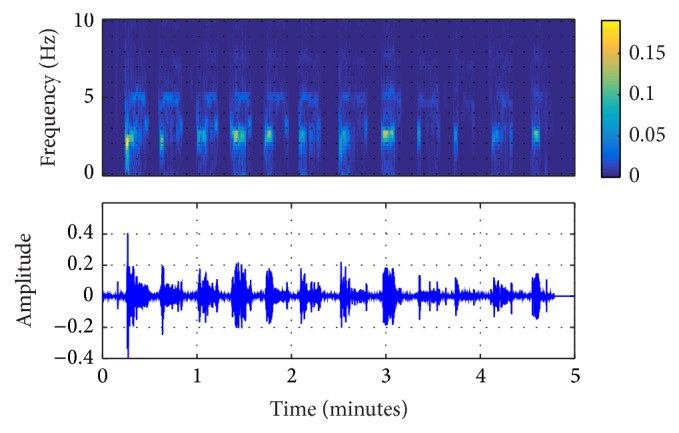
EEMD-extracted CPR and the time-frequency information of IMF 3.

**Figure 4:**
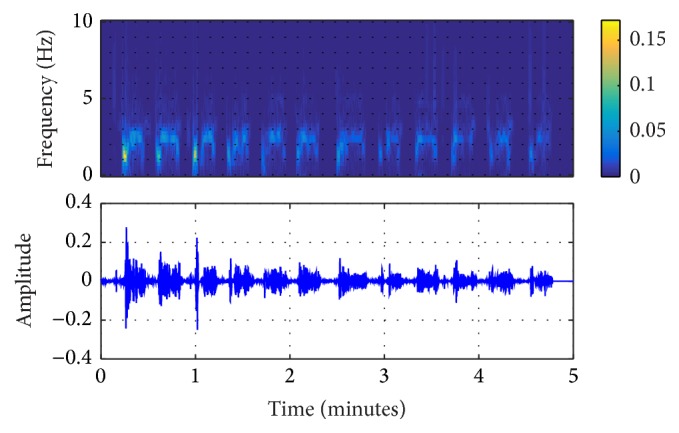
EEMD-extracted CPR and the time-frequency information of IMF 4.

**Figure 5:**
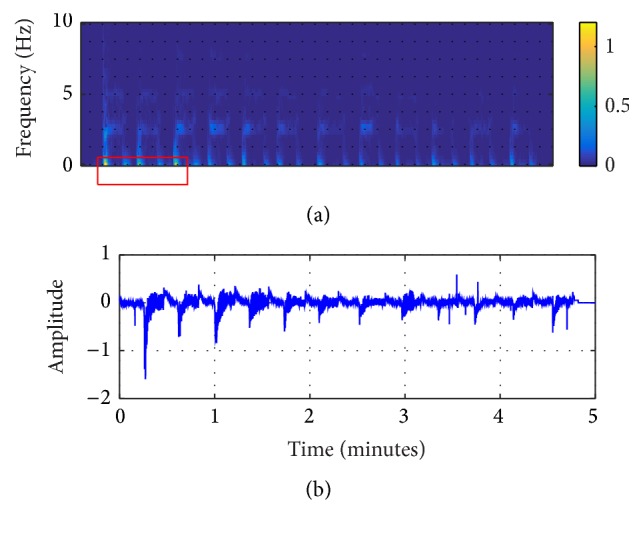
Raw signal from AED machine. (a) Time-frequency result; (b) the raw signal.

**Figure 6:**
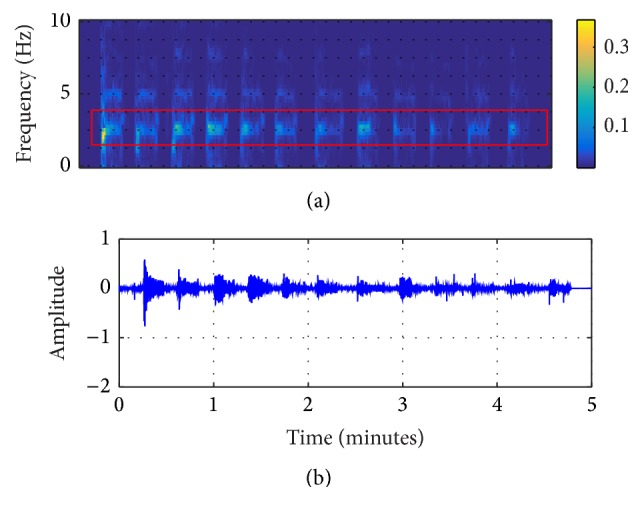
EEMD-reconstructed CPR signal. (a) Time-frequency result; (b) the reconstructed signal.

In this study, the original ECG logged from the AED machine was filtered by the EEMD algorithm, shown in Figures 2 [Fig fig3]–4. From these figures, it can be seen that IMF 2 to IMF 4 are relatively similar to the CPR pattern having the dominant frequency as described in previous study conducted by Lo et al. [27]. Figures 5 and 6 also show the time-frequency evaluation; this shows the differences between the raw ECG and the reconstructed-CPR signal after the EEMD filter by combining the CPR-related IMFs. Figures 5(a) and 6(a) give the information about the time-frequency information. For Figure 5(a), the dominant signal occurs mostly below the CPR frequency ranges, lower than 0.5 Hz, indicated by the red area. Meanwhile, for Figure 6(a), after the EEMD filter, the dominant frequency shifts to the range of 2 Hz to 4 Hz, indicated by the red square. This filter also automatically reduces the baseline noise of the signal that can be seen by Figures 5(b) and 6(b).

**Figure 7:**
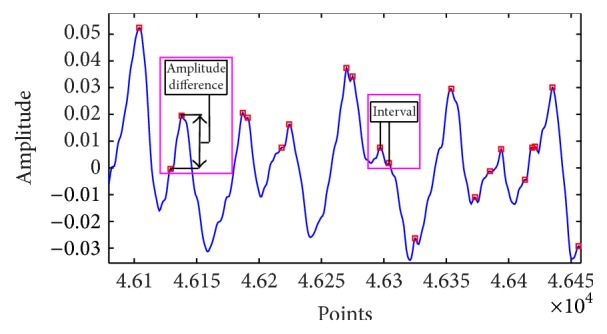
CPR IMFs maxima information evaluation.

All the maxima points are detected from the reconstructed IMFs that have the CPR frequency, by evaluating the changing of the slopes from positive to negative as shown in Figure 7. Furthermore, the maxima points are evaluated to obtain the maxima interval (*I*) and maxima amplitude differences (dA) from the IMF-combined CPR, shown in Figure 7. Furthermore, both signals, *I* and dA, are estimated by utilizing SE, CI, and DFA.

Evaluation of the results of the 951 ECGs from patients of non-trauma OHCAs with a non-shock-able rhythm using a threshold of 60 years of age is shown in Table 1. A subgroup analysis is performed which begins for patients greater than 60 years of age: of this category 579 patients died and 116 patients survived. The mean SE value is 1.91 ± 0.58 and 1.87 ± 0.56 for dead and surviving patients, respectively (*p* > 0.05). CI for patients who died is 13.26 ± 4.46 and for those who survived is 13.48 ± 4.67 (*p* > 0.05). The DFA evaluation is 0.86 ± 0.145 for patients who have died and 0.833 ± 0.136 for those who have survived (*p* > 0.05).

A further subgroup analysis is performed for patients under 60 years of age. The total number of the patients for this class is less than half of the number of the patients older than 60 years. The observed SE is 1.86 ± 0.61 and 1.81 ± 0.6, respectively, for the patients who have died compared to those who have survived (*p* > 0.05). The CI is 13.12 ± 4.9 and 12.03 ± 4.26, respectively, for patients who have died compared to those who have survived. The DFA is 0.839 ± 0.15 and 0.845 ± 0.12, respectively, for patients who died and survived and also not significantly different.

On assessment of the amplitude difference, for patients aged 60 or over, patients who died had a mean SE value of 0.22 ± 0.236 and for the patients who have survived, the results are 0.226 ± 0.244 (*p* > 0.05). CI for patients who have died is 1.23 ± 1.24 versus 1.195 ± 1.184 for those who have survived (*p* > 0.05).

For cases of the category of age of less than 60 years, the SE has 0.2 ± 0.23 and 0.24 ± 0.16, respectively, of patients who have died and are alive and has no significant differences. The CI has 0.983 ± 1.03 and 1.378 ± 1.173, respectively, for those who died and survived; this case is significantly different (*p* < 0.05). The DFA case creates 0.105 ± 0.168 and 0.107 ± 0.098 (*p* > 0.05).

In terms of the relationship of this result to the OHCA for the future applications, the focus is the automated CPR machine. According to a study by Steen et al., the automated CPR machine was very advantageous in performing the chest compression during transportation way [33]. The automated CPR also produced better pressure of end tidal carbon dioxide (*P*_ET_CO_2_) [34] and cortical blood flow [35] compared to the manual CPR. However, a study with 4471 patients conducted by Perkins et al., with the consistent rate and depth, shows the automated CPR is not significantly different from the manual CPR with the main outcome being the survival rate after 30 days of OHCA [36]. In another study by Smekal et al., evaluated automated and manual CPR for 75 and 73 patients, respectively, also provided no significant difference [37]. Also, Hallstrom et al. investigated a total of 554 and 517 for automated CPR and manual CPR, respectively. This study found that the automated CPR reduced the survival and made the neurological outcome worse [38]. The controversial results of the previous studies of automated and manual CPR may be due to the consistent amplitude of the automated CPR machines. By referring to our study's results, not that consistent depth for the CPR amplitude, which can be implemented into the CPR machine for the future tests, may increase the survival rate.

## 4. Conclusions and Future Work

This study evaluates a total of 951 of the non-shock-able patient ECGs, using the ensemble empirical mode decomposition filtering and utilizing nonlinear approaches. The IMF-combined CPR maxima interval and the amplitude are evaluated. For most of the observations, there were no statistically significant differences observed. However, in the evaluation of CI for the maximal amplitude, a statistically significant difference was observed.

Based on the results, it can be concluded that for patients who are less than 60 years of age a higher survival rate was observed and was associated with more complexity in CPR amplitude differences. This result can have information that the automated CPR machine with the dynamic force may be a consideration.

This study has several limitations. Namely, when the noise interference occurred at the same frequency range of the CPR IMFs, they were included in the evaluation. This may somewhat affect the observations, especially for the slope evaluation. Furthermore, there were far more observations for patients who died than for those who survived.

For future study, the application of the advanced time-domain filter may be applied to purify the unfiltered noise on the frequency domain filter.

## Figures and Tables

**Table 1:** The statistical evaluation of the CPR IMFs result.

Evaluation	Age	Feature	Status	Mean	Standard deviation	*p* value (*p* < 0.05)
Interval	≥60 (579,116)	SE	Died	1.91	0.58	0.556
			Survival	1.87	0.56	
		CI	Died	13.26	4.46	0.62
			Survival	13.48	4.67	
		DFA	Died	0.86	0.145	0.06
			Survival	0.833	0.136	

Interval	<60 (215,41)	SE	Died	1.86	0.61	0.575
			Survival	1.81	0.6	
		CI	Died	13.12	4.9	0.234
			Survival	12.03	4.26	
		DFA	Died	0.839	0.15	0.825
			Survival	0.845	0.12	

Amplitude	≥60 (579,116)	SE	Died	0.22	0.236	0.825
			Survival	0.226	0.244	
		CI	Died	1.23	1.24	0.781
			Survival	1.195	1.184	
		DFA	Died	0.115	0.126	0.215
			Survival	0.099	0.1165	

Amplitude	<60 (215,41)	SE	Died	0.2	0.23	0.28
			Survival	0.24	0.16	
		*CI*	*Died*	*0.983*	*1.03*	***0.028*** ^*∗*^
			*Survival*	*1.378*	*1.173*	
		DFA	Died	0.105	0.168	0.912
			Survival	0.1077	0.0983	

*Note*. SE means sample entropy, CI complexity index, and DFA detrended fluctuation analysis; ^*∗*^significant different parameter.
